# Effects of Steam Sterilization on the Properties of Stimuli-Responsive Polymer-Based Hydrogels

**DOI:** 10.3390/gels9050385

**Published:** 2023-05-06

**Authors:** Inês Ferreira, Ana Camila Marques, Paulo Cardoso Costa, Maria Helena Amaral

**Affiliations:** 1 UCIBIO-Applied Molecular Biosciences Unit, MEDTECH-Medicines and Healthcare Products, Laboratory of Pharmaceutical Technology, Department of Drug Sciences, Faculty of Pharmacy, University of Porto, R. Jorge de Viterbo Ferreira 228, 4050-313 Porto, Portugal; up201904788@edu.ff.up.pt (I.F.); pccosta@ff.up.pt (P.C.C.); 2 Associate Laboratory Institute for Health and Bioeconomy—i4HB, Faculty of Pharmacy, University of Porto, R. Jorge Viterbo Ferreira 228, 4050-313 Porto, Portugal

**Keywords:** hydrogels, smart polymers, sterilization

## Abstract

Hydrogels based on stimuli-responsive polymers can change their characteristics in response to small variations in environmental conditions, such as temperature, pH, and ionic strength, among others. In the case of some routes of administration, such as ophthalmic and parenteral, the formulations must meet specific requirements, namely sterility. Therefore, it is essential to study the effect of the sterilization method on the integrity of smart gel systems. Thus, this work aimed to study the effect of steam sterilization (121 °C, 15 min) on the properties of hydrogels based on the following stimuli-responsive polymers: Carbopol^®^ 940, Pluronic^®^ F-127, and sodium alginate. The properties of the prepared hydrogels—pH, texture, rheological behavior, and sol-gel phase transition—were evaluated to compare and identify the differences between sterilized and non-sterilized hydrogels. The influence of steam sterilization on physicochemical stability was also investigated by Fourier-transform infrared spectroscopy and differential scanning calorimetry. The results of this study showed that the Carbopol^®^ 940 hydrogel was the one that suffered fewer changes in the studied properties after sterilization. By contrast, sterilization was found to cause slight changes in the Pluronic^®^ F-127 hydrogel regarding gelation temperature/time, as well as a considerable decrease in the viscosity of the sodium alginate hydrogel. There were no considerable differences in the chemical and physical characteristics of the hydrogels after steam sterilization. It is possible to conclude that steam sterilization is suitable for Carbopol^®^ 940 hydrogels. Contrarily, this technique does not seem adequate for the sterilization of alginate or Pluronic^®^ F-127 hydrogels, as it could considerably alter their properties.

## 1. Introduction

In recent decades, hydrogels (HGs) have been widely used in the pharmaceutical and biomedical fields due to their hydrophilicity, biocompatibility, and adjustable mechanical properties similar to those of body tissue components [1].

HGs are three-dimensional (3D) cross-linked polymeric networks capable of retaining large amounts of water and biological fluids [2]. This high affinity for water can be attributed to the presence of hydrophilic groups, such as OH, CONH, CONH_2_ and SO_3_H [3,4]. These systems, especially HGs based on polymers responsive to external stimuli, proved to be important drug carriers [5]. Stimuli-responsive polymers, also known as smart polymers, undergo reversible changes in their microstructure and physicochemical properties in response to small changes in the surrounding environment, such as temperature, light, magnetic field, pH, ionic strength, and the presence of biomolecules, among others. The physical-chemical changes that occur in the structure of polymers can be in its physical state, in its form and solubility, in its conductivity and/or in its hydrophilic/hydrophobic balance [6].

Indeed, the mechanical properties of HGs are essential for their use as pharmaceutical dosage forms and as scaffolds for tissue engineering. Changing the degree of crosslinking of the HGs allows it to obtain desirable mechanical properties. Therefore, there is an optimal degree of crosslinking to obtain a HG that is relatively resistant but at the same time flexible [7].

In this work, only HGs made of polymers responsive to temperature, pH and ionic strength will be considered.

### 1.1. Temperature-Responsive Polymers

One of the most important characteristics of temperature-responsive polymers is the critical solution temperature, which is defined as the temperature at which the polymeric system undergoes phase separation. There are two types of temperature-responsive polymers, depending on whether they undergo gelling on cooling below the upper critical solution temperature (UCST) or by heating above the lower critical solution temperature (LCST). Polymers with a LCST are monophasic (in solution) below the critical solution temperature. On the contrary, when the temperature is higher than the critical solution temperature, this polymer becomes insoluble, with phase separation and formation of a gel [8]. An example of an LCST-type polymer is Poloxamer 407, usually designated by the trade name Pluronic^®^ F-127, a non-ionic triblock copolymer consisting of two monomeric units of ethylene oxide and propylene oxide, which is amphiphilic in an aqueous medium. At low temperatures Pluronic^®^ F-127 is in liquid state, due to the presence of hydrogen bonds between water and their hydrophilic ethylene oxide chains (PEO). However, when temperature increases, the poloxamer solution suffers a sol-gel transition as a consequence of the hydrogen bonds breaking and the interactions among their hydrophobic propylene oxide chains (PPO). This is called thermoreversible gelation. The non-covalent interactions formed in this stage return to sol state after the temperature decreases [9,10]. This polymer is widely used in the development of injectable HGs because it exhibits a sol-gel phase transition near the body temperature [5]. 

### 1.2. pH-Responsive Polymers

Regarding pH-responsive polymers, these have ionizable groups attached to the main chain that accept or donate protons depending on the pH of the external environment. If the ionizable groups are weak acids, such as carboxyl groups, the polymers are considered polyacids and give rise to anionic HGs. When the groups are weak bases, namely the amine groups, they are polybases and give rise to cationic HGs [5,11].

An example of a widely used pH-responsive polymer is polyacrylic acid and its derivatives. Carbomers designated by the trade name Carbopol^®^, are high-molecular-weight anionic synthetic polymers, composed of acrylic acid cross-linked with sucrose allylic ethers or pentaerythrityl allylic ethers [12]. Aqueous dispersions of these polymers can be prepared and after neutralization with bases, such as triethanolamine, clear HGs are obtained.

### 1.3. Ionic Strength Responsive Polymers

Ionic strength sensitive polymers belong to the in situ gelling materials mainly used for ocular drug delivery. In this case, gelation of the instilled solution is triggered by a change in ionic strength [13]. Polymers that contain ionizable groups in their structure are responsive to ionic strength. HGs are formed by ionic cross-linking in response to changes in electrolyte concentration, whereby the sol-gel phase transition is triggered by ionic interactions between negative charges in the polymer and cations in the surrounding medium [14].

A representative example of an ionic-strength responsive polymer is alginate, which is a high-molecular-weight unbranched binary copolymer of 1–4 glycosidically linked β-D-mannuronic and α-L-glucuronic acid monomers. The high acid content present in its structure allows alginic acid to spontaneously gel in the presence of divalent cations such as calcium ions [13].

Before being used in tissue engineering and in the pharmaceutical area, namely in pharmaceutical dosage forms for parenteral and ophthalmic administration, HGs must be sterilized. However, the sterilization process may have limitations due to the nature of HGs, their 3D network architecture, and water [1]. The sterilization method should not alter the HG’s mechanical, functional and chemical properties.

Conventional terminal methods for sterilizing HGs include ethylene oxide, gamma radiation, and steam sterilization. Studies revealed that these sterilization methods might alter the HG’s properties. When the materials cannot withstand high temperatures or radiation, gases such as ethylene oxide are typically used [15]. Its mechanism of action involves the irreversible alkylation of cellular components. Despite the efficiency and good penetration of ethylene oxide, it can modify mechanical properties, accelerate polymer degradation, and leave toxic residues if the gas and its derivates are not completely removed [16,17]. Sterilization can also be achieved by exposing the HG to ionizing radiation, specifically cobalt-60 gamma radiation, which breaks DNA and RNA directly or generates reactive oxygen species (ROS) that damage other important biomolecules [18]. Although gamma radiation has strong penetrability with no residual toxicity, this method takes hours to days and is associated with changes in the HG network, namely chain scission or further crosslinking [16,19]. Steam sterilization combines high temperature and high humidity under pressure to destroy microorganisms through the irreversible denaturation of proteins. This process occurs without toxic waste in an autoclave, generally at temperatures between 121 and 130 °C for short periods (e.g., 15–20 min). The presence of water vapor has been shown to induce the melting or hydrolytic degradation of some polymers [20]. Still, steam was preferred in this study as it is the most employed, straightforward, and low-cost sterilization method.

In this sense, the main objective of this study was to evaluate the effect of steam sterilization at 121 °C for 15 min on the properties of HGs based on polymers responsive to temperature, pH, and ionic strength: Pluronic^®^ F-127 (PF127), Carbopol^®^ 940 (C940), and sodium alginate (SA). The pH, texture, rheological behavior, and sol-gel transition were evaluated in order to ascertain the differences between HGs sterilized by steam and those not submitted to sterilization. Fourier-transform infrared (FT-IR) spectroscopy and differential scanning calorimetry (DSC) were also performed to investigate the influence of steam sterilization on the HGs’ physicochemical stability.

## 2. Results and Discussion

### 2.1. Preparation of the Hydrogels

In this work, stimuli-responsive HGs were prepared as summarized in Table 1.

Afterward, the effects of steam sterilization on their properties (rheological, mechanical, pH, and sol-gel transition) were studied at the following time points: one week (T0), two weeks (T1) and one month (T2) after preparation. The polymer concentration was chosen based on that used successfully in the literature [21,22,23,24,25]. After each polymer was fully dispersed in purified water containing a preservative (Fenonip^®^), three different HGs were obtained. All formulations were transparent except SA HGs, which appeared translucent. Both PF127 HGs and SA HGs corresponded to low viscosity liquids (sol state). Steam sterilization did not change that appearance.

### 2.2. Characterization of the Hydrogels

#### 2.2.1. Rheological Characterization

**Figure 1:**
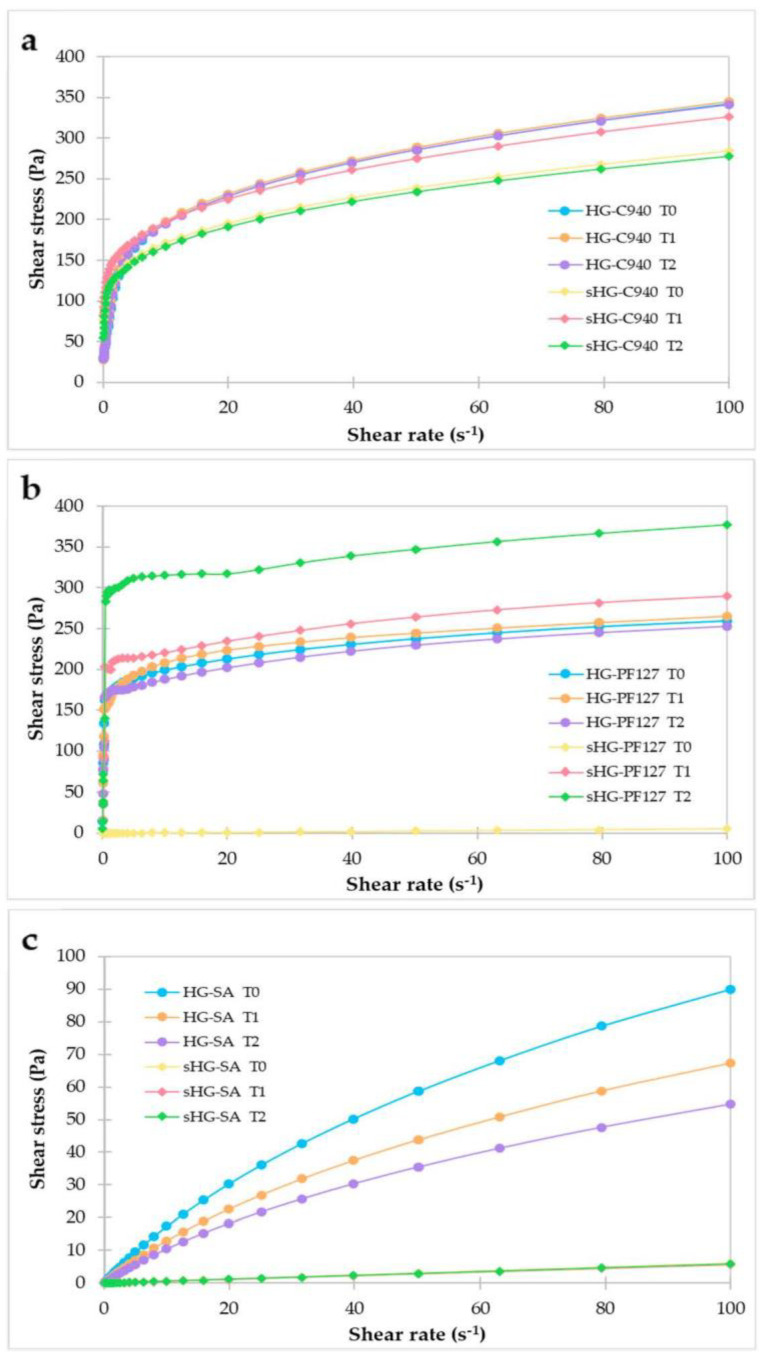
Rheograms of sterilized (sHG) and non-sterilized (HG) hydrogels based on Carbopol^®^ 940 (C940) (**a**), Pluronic^®^ F–127 (PF127) (**b**), and sodium alginate (SA) (**c**) obtained at one week (T0), two weeks (T1) and one month (T2) after preparation.

Rheology is very useful to understand the flow behavior and structural changes of HGs upon steam sterilization. Figure 1 shows the graphs representing the flow properties of HGs as a function of shear rate (i.e., rheograms).

The C940 HGs presented a non-Newtonian, shear-thinning behavior with a yield stress, meaning that after this yield value is achieved, the systems start to flow, and their apparent viscosity decreases with increasing shear rate [26]. The shear stress vs. shear rate graph (Figure 1a) indicated shear thinning as the shear rate increases faster than the shear stress, resulting in a curve concave to the horizontal axis [27]. Considering the ease of application and stability of HGs during storage, this behavior is the most appreciated [28]. The non-sterilized C940 HG (HG-C940) exhibited higher viscosity than the sterilized one (sHG-C940), which remained identical for a month. The viscosity values of sHG-C940 became closer to those of HG-C940 at T1 but then returned to the initial values a couple of weeks later.

As can be seen in Figure 1b, the non-sterilized PF127 HG (HG-PF127) was a non-Newtonian fluid with shear-thinning behavior and viscosity values quite similar over 30 days after preparation. Interestingly, the sterilized PF127 HG (sHG-PF127) started exhibiting a Newtonian behavior since the viscosity was independent of the shear rate. Then, a shift in the flow type from Newtonian to shear-thinning was observed at T1, suggesting that PF127 HGs cannot reach gelation right after steam sterilization and probably need a longer maturation between autoclaving and testing. In the second week and one month after preparation, sHG-PF127 experienced an increase in viscosity compared to HG-PF127. It is assumed that the steam sterilization reduced the sHG-PF127 water content due to evaporation, causing its polymer weight fraction to increase and consequently its viscosity [29].

Regarding the non-sterilized SA HG (HG-SA), a shear-thinning behavior was also identified, but viscosity decreased somewhat over 30 days (Figure 1c). However, the exposure to steam induced a change in the flow behavior of the sterilized SA HG (sHG-SA) to a Newtonian fluid, as well as a sharp reduction in viscosity. The latter is consistent with previous works reporting that autoclaving significantly reduced the alginate molecular weight and, in turn, the HG viscosity [30,31].

**Figure 2:**
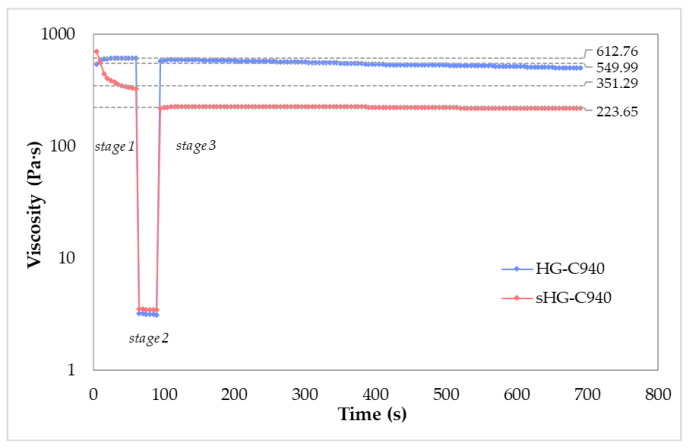
Three-step shear test for evaluating the thixotropy of sterilized (sHG) and non-sterilized (HG) hydrogels based on Carbopol^®^ 940 (C940).

According to the results above, C940 HGs were selected for the study of thixotropy because these systems were the least impacted by sterilization. A three-step test applying low (0.1 s^−1^), high (100 s^−1^) and low (0.1 s^−1^) shear rates for a given time is the best way to evaluate time-dependent changes in viscosity. When the stress was removed in stage 3, HG-C940 recovered 90% of its original viscosity (612.76 vs. 549.99 Pa·s) after 600 s, while the recovery rate of sHG-C940 in the same period was 64% (351.29 vs. 223.65 Pa·s) (Figure 2). Therefore, HG-C940 is not very thixotropic, unlike the sterilized sample that appeared to take some time for the structure to rebuild and become thick again.

#### 2.2.2. Texture Analysis

**Figure 3:**
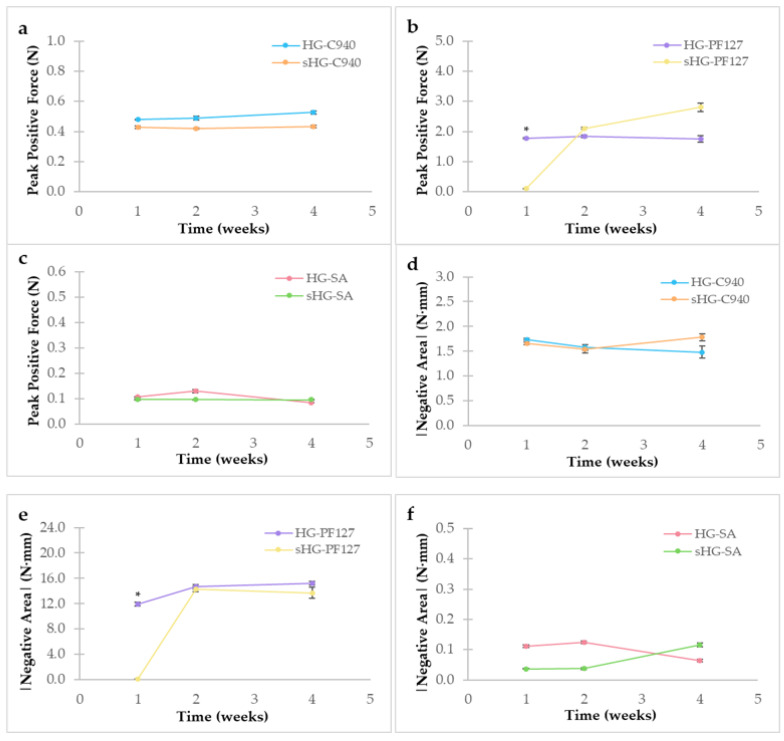
Firmness (**a**–**c**) and adhesiveness (**d**–**f**) of sterilized (sHG) and non-sterilized (HG) hydrogels based on Carbopol^®^ 940 (C940) (**a**,**d**), Pluronic^®^ F–127 (PF127) (**b**,**e**), and sodium alginate (SA) (**c**,**f**) one, two and four weeks after preparation. Error bars represent mean ± SD (*n* = 3). * *p* < 0.05 denotes a significant difference between the sterilized and non-sterilized hydrogels by independent samples Kruskal–Wallis’ test.

The mechanical properties of the HGs were tested at three time points (one, two and four weeks after preparation) using a texture analyzer in compression mode (5 kg load cell, P/25L probe, 0.049 N trigger force). The Exponent software used the peak positive force and the negative area absolute value, based on the obtained force vs. distance graph, to characterize firmness (Figure 3a–c) and adhesiveness (Figure 3d–f), respectively.

Similar results in each parameter were observed for HG-C940 and sHG-C940 during the study. The same went for non-sterilized and sterilized SA HGs. Moreover, SA HGs showed lower firmness and adhesiveness than the other formulations, which can be related to their lowest viscosity at 25 °C [32]. Apart from sHG-PF127 one week after preparation, PF127 HGs presented the highest firmness and adhesiveness. Given the temporary loss of its gelation properties upon autoclaving, the first results of firmness (Figure 3b) and adhesiveness (Figure 3e) for sHG-PF127 were significantly lower than those for HG-PF127 (*p* = 0.039 and *p* = 0.006, respectively). No statistical difference between sterilized and non-sterilized HGs could be found one month after preparation.

#### 2.2.3. pH Measurement

**Figure 4:**
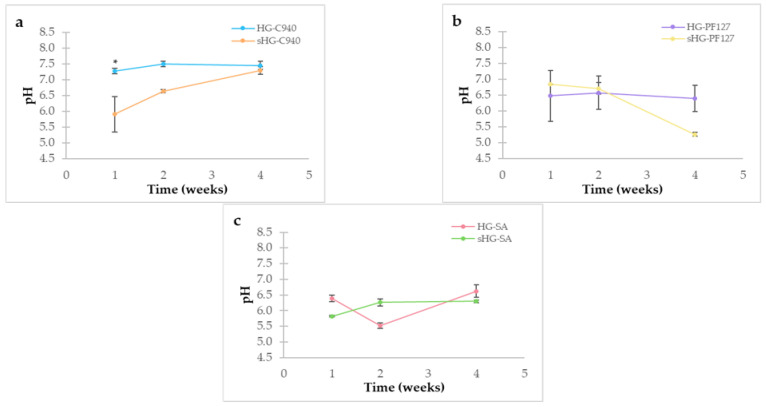
pH of sterilized (sHG) and non-sterilized (HG) hydrogels based on Carbopol^®^ 940 (C940) (**a**), Pluronic^®^ F–127 (PF127) (**b**), and sodium alginate (SA) (**c**) one, two and four weeks after preparation. Error bars represent mean ± SD (*n* = 3). * *p* < 0.05 denotes a significant difference between the sterilized and non-sterilized hydrogels by independent samples Kruskal–Wallis’ test.

Measuring the pH of HGs is critical to avoid irritation at the site of application. Figure 4 shows the results obtained one, two and four weeks after preparation.

Compared to other formulations, the pH values of C940 HGs were relatively higher as triethanolamine was added dropwise to complete the gelation process. Accordingly, the pH of C940 HGs was close to neutrality, whereas PF127 HGs and SA HGs were found to be slightly acidic. In general, sterilized HGs showed a higher variation in pH values than non-sterilized HGs. Although steam sterilization significantly lowered the pH value of sHG-C940 at first (*p* = 0.005), this formulation reached its initial value three weeks later. Additionally, there were no significant changes in pH between HG-SA and sHG-SA or over time (*p* = 0.491).

It is noteworthy that the pH values of the sterilized HGs ranged from 5.3 to 7.3, thereby being around physiologically relevant pH values. Furthermore, this pH range is suitable for ophthalmic (pH 5.0 and 8.5) [33] or nasal (pH 5.5–6.5) administration [34].

#### 2.2.4. Sol-Gel Transition

The evaluation of sol-gel transition was only carried out in the case of PF127 HGs. Oscillatory shear rheology was used to study the viscoelastic properties of PF127 HGs, measuring their elastic (or storage) modulus (G′) and viscous (or loss) modulus (G″). Whereas the former gives information about the stored elastic energy, the latter describes the energy dissipated as heat [35].

**Figure 5:**
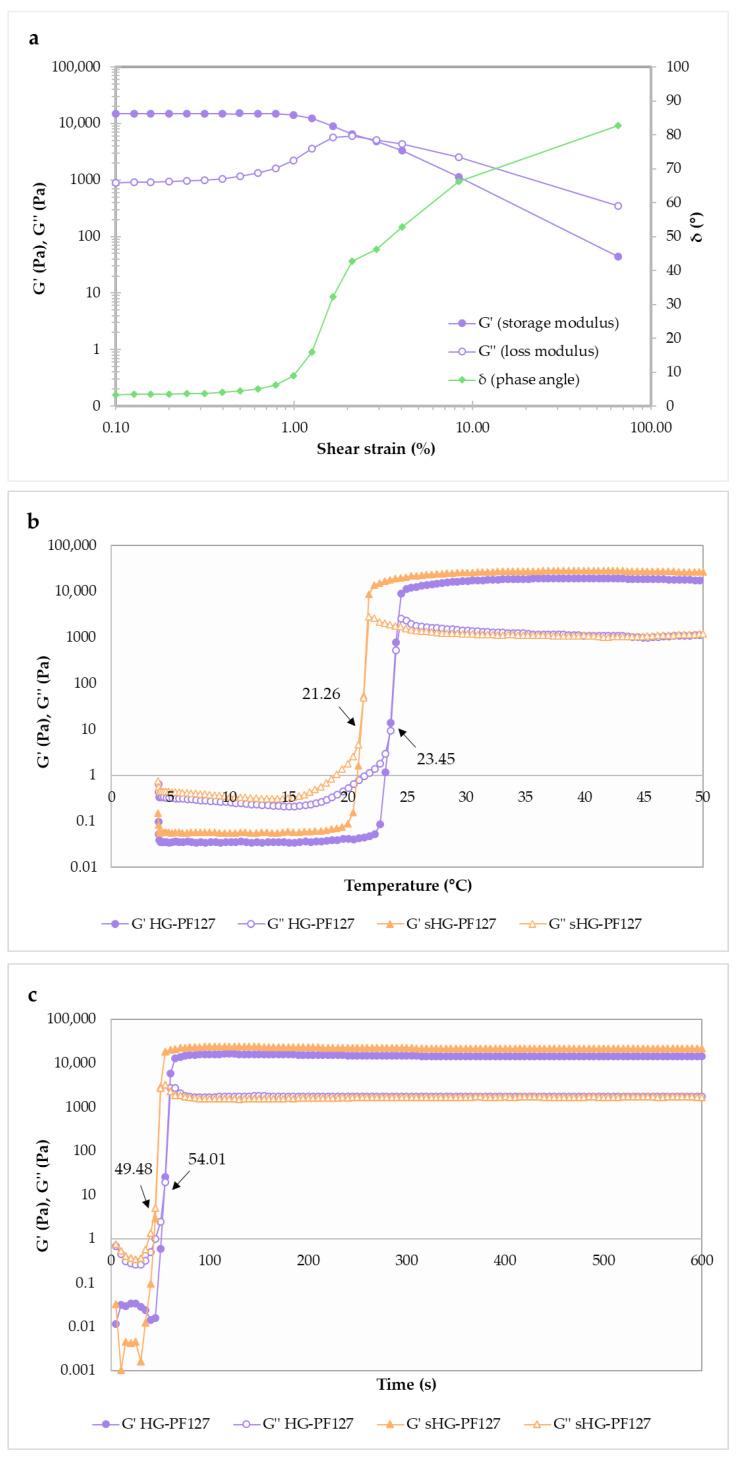
Oscillatory rheological measurements of sterilized (sHG) and non-sterilized (HG) hydrogels based on Pluronic^®^ F–127 (PF127): amplitude sweep (**a**), temperature sweep (**b**), and time sweep (**c**). G′, G″ corresponds to elastic and viscous moduli and δ to phase angle.

The strain sweep (amplitude sweep) test (0.1–100%, 1 Hz, 25 °C) was performed first to define the linear viscoelastic (LVE) region of HG-PF127, where both viscoelastic moduli (G′ and G″) are independent of the applied strain amplitude [36]. Figure 5a illustrates that G′ and G″ run almost parallel without a crossover point up to 1% strain. In that linear region, G’ was always greater than G″ and the phase angle (δ) was less than 45 °C, underlining the solid-like behavior of the sample [37,38]. A strain of 0.5% was selected for subsequent sweeps to prevent the network breakdown of PF127 HGs and ensure that their microstructural properties would be considered. The gelation point of PF127 HGs can be determined as a function of temperature or time. Hence, testing involving temperature ramps from 4 to 50 °C (5 °C/min rate) and time sweeps over 600 s at 25 °C was performed.

As shown in Figure 5b, there was a prevalence of G″ at lower temperatures, meaning that both sterilized (sHG-PF127) and non-sterilized (HG-PF127) systems behave like a viscous liquid (sol state) initially. Then, a thermally induced sol-gel transition was observed when the G″ values surpassed those of G′ (G′/G″ crossover). The gelation temperature (T_sol-gel_) was calculated by interpolation from a graph of G’, G″ and δ vs. temperature (data not shown), considering that G′ and G″ cross at δ = 45° [39]. The results indicated that steam sterilization slightly reduced T_sol-gel_ from 23.45 to 21.26 °C, which is in line with the observation of Beard et al. [29]. It is established that the higher the poloxamer concentration, the lower the gelation temperature [40]. Therefore, the T_sol-gel_ of sHG-PF127 can be explained by the increase in polymer weight fraction arising from water evaporation during autoclaving. Nevertheless, since both T_sol-gel_ values are too low for physiological applications, the combination of PF127 with Poloxamer 188 is recommended [41].

Gelation should be fast to avoid dilution with physiological fluids and burst release from the gel, but not to the point it is too fast to form uniform structures [42,43]. The time-dependent changes in G′ and G″ moduli during gelation at 25 °C are represented in Figure 5c. Gelation time is defined as the time after which G′ becomes higher than G″ at a constant temperature and herein was interpolated by using a G′, G″ and δ vs. time graph (data not shown). The gelation of sHG-PF127 at 25 °C occurred in 49.48 s, while HG-PF127 took a few more seconds to gel (54.01 s).

The overall results show a trend of decreasing gelation temperature/time when PF127 was at higher concentrations, namely after steam sterilization.

The gel-sol transition at higher temperatures is not yet described as precisely as the sol-gel transition, but it can be explained by the shrinking of the upper parts of POE in micelles, related with the impact of temperature on POE solubility, and interaction of this with the POP core [44].

#### 2.2.5. Fourier-Transform Infrared (FT-IR) Spectroscopy 

**Figure 6:**
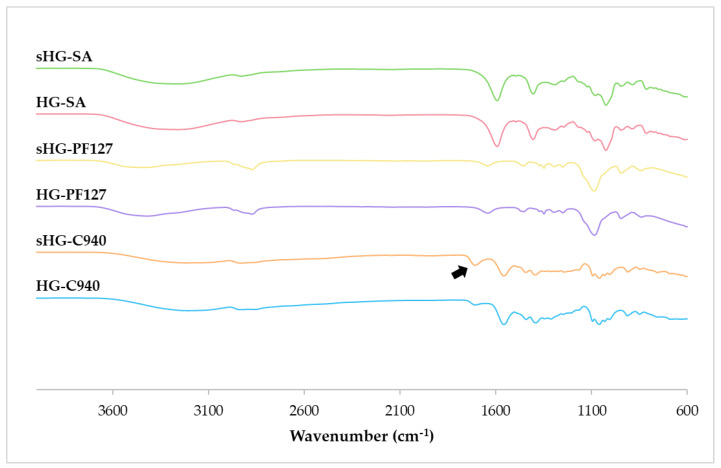
Fourier-transform infrared (FT-IR) spectra of sterilized (sHG) and non-sterilized (HG) hydrogels based on Carbopol^®^ 940 (C940), Pluronic^®^ F–127 (PF127), and sodium alginate (SA).

Chemical characterization of sterilized and non-sterilized HGs was performed by FT-IR spectroscopy, as illustrated in Figure 6.

In the FT-IR spectrum of HG-C940, the peak at 1702 cm^−1^ corresponded to C=O stretching, while absorption at 1546, 1442 and 1394 cm^−1^ indicated C–O/O–H. Additionally, the peak at 834 cm^−1^ confirmed the plane bending of C=CH of the acrylate [45]. Following autoclaving, all the signals were still visible, but the peak associated with carbonyl stretching became more pronounced and shifted to 1698 cm^−1^ (black arrow). Contrastingly, spectral overlapping was observed for PF127 and SA HGs. The spectrum of HG-PF127 revealed C–H stretching, in-plane O–H bending, and C–O stretching at 2868 cm^−1^, 1348 cm^−1^, and 1086 cm^−1^, respectively [46]. Moreover, the bending vibration of –CH bonds was observed between 1720 and 1416 cm^−1^, and C–O–C stretching was registered at 1294 and 1246 cm^−1^ [47,48]. The above-mentioned typical bands of PF127 also appeared in the spectrum of sHG-PF127. The spectra of both SA HGs showed a broad absorption band between 3650 and 3000 cm^−1^, which was assigned to O–H stretching. Other peaks of SA at 1594 cm^−1^ (C–O asymmetric stretching), 1404 cm^−1^ (C–O symmetric stretching), 1024 cm^−1^ (C–O–C stretching), 948 cm^−1^ (symmetric stretching in the pyranose ring), 884 cm^−1^ (C–O–C symmetric stretching in 1,4-glycosidic bonds), and 812 cm^−1^ (flexion vibration in guluronic and mannuronic acid units) were also identified [49]. 

Based on these findings, there was no noticeable difference in the functional groups of PF127 and SA HGs after steam sterilization. Interestingly, while C940 HG suffered fewer changes in texture and rheological properties, its chemical structure seemed to be somewhat affected by autoclaving.

#### 2.2.6. Differential Scanning Calorimetry (DSC)

**Figure 7:**
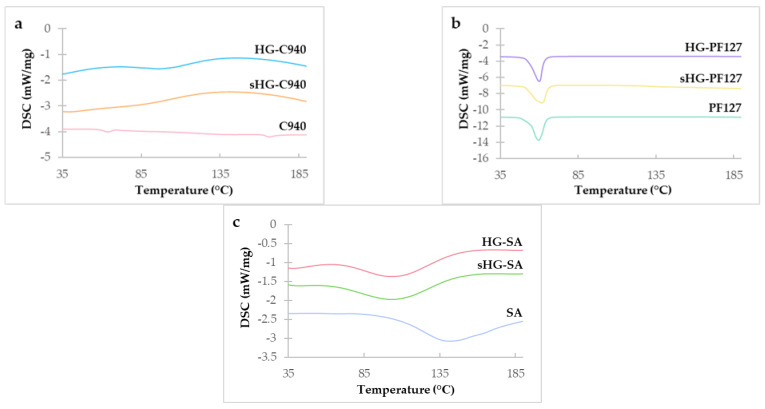
DSC thermograms of Carbopol^®^ 940 (C940) (**a**), Pluronic^®^ F–127 (PF127) (**b**), and sodium alginate (SA) (**c**) and their respective sterilized (sHG) and non-sterilized (HG) hydrogels.

The obtained DSC thermograms are presented in Figure 7. According to Figure 7a, C940 exhibited two endothermic peaks at 63.9 °C and 166.3 °C, with the former being associated with the polymer’s glass transition [50].

A weak and broad endothermic peak at around 100 °C in HG-C940 was attributed to the evaporation of water remaining in the sample despite drying before analysis. This was not observed in the thermogram of sHG-C940, probably because autoclaving already reduced its water content [16]. In Figure 7b, the endothermic peak of each thermogram corresponds to the melting point of PF127 (Tm range: 52–57 °C) [51]. Although the location of the peak remains virtually the same, the melting enthalpy (i.e., the integrated area under the peak) of PF127 (∆H = 139.9 J/g) slightly decreased after polymer hydration (∆H = 134.6 J/g) and even more so with HG sterilization (∆H = 132.1 J/g). We hypothesize that HG-PF127 required less energy for polymer melting due to residual water weakening polymer-polymer interactions, while sterilization might have caused a tiny fraction of the polymer to degrade. The DSC thermogram of SA (Figure 7c) was in reasonable agreement with the reported data [52], and both HGs displayed an endothermic peak consistent with some water evaporation. Moreover, the DSC curves of HG-SA and sHG-SA follow a similar pattern, suggesting that steam sterilization did not appreciably alter the physical properties of this polymer.

## 3. Conclusions

For in vivo applications of HGs, sterilization reduces the risk of infection and inflammation. Thus, the selection of an appropriate sterilization method is important in the case of stimuli-responsive HGs used for biomedical and pharmaceutical purposes.

With this study it was possible to conclude that steam sterilization is an adequate method for the C940 HG. However, in the case of SA HG, this method of sterilization irreversibly alters its rheological properties. 

As PF127 is a temperature-responsive polymer, it was expected that the respective HG would suffer a greater impact after steam sterilization. In fact, after sterilization, the PF127 HG initially behaved like a Newtonian material. However, after 15 days, it exhibited a rheological behavior equal to that of the non-sterile HG (non-Newtonian, shear thinning behavior), but with a higher viscosity. In addition, the temperature and gelling time of PF127 decreased slightly after sterilization. 

Steam autoclaving had a certain impact on the rheological properties of PF127-based HGs. Thus, as verified by Burak et al. [44], it can be concluded that a longer duration of autoclaving with a lower temperature (e.g., 105 °C for 30 min) allows the acquisition of HGs with properties more similar to non-sterilized ones.

## 4. Materials and Methods

### 4.1. Materials

C940 was purchased from J. Vaz Pereira (Benavente, Portugal), while PF127 ((C3H6O·C2H4O)_x_) and SA were obtained from Sigma-Aldrich (St. Louis, MO, USA) and Acros Organics BVBA (Geel, Belgium), respectively. Fenonip^®^ was purchased from Acofarma (Madrid, Spain). Triethanolamine was purchased from Fagron (Barcelona, Spain). Purified water was obtained from a Milli-Q Direct-Q^®^ Water Purification System (Merck KGaA, Darmstadt, Germany).

### 4.2. Preparation of the Hydrogels

The composition of the different HGs is presented in Table 1. To prepare the HGs, the preservative Fenonip^®^ was first added to purified water, hence preventing microbial growth. C940 was dispersed in this aqueous solution, which was then neutralized with triethanolamine. The other polymers were dispersed in water containing Fenonip^®^ using a mechanical stirrer (Heidolph RZR 2041, Heidolph Instruments GmbH & Co. KG, Schwabach, Germany) at 750 rpm for 1.5 h. In the case of PF127, the beaker was kept in an ice bath during stirring, according to the “cold” method [53]. Finally, all HGs were stored in the refrigerator at 4 °C.

The sterilized HGs were prepared as previously described, stored at 4 °C for 48 h, and then subjected to steam sterilization at 121 °C for 15 min in an autoclave (Uniclave 88, AJC, Cacém, Portugal).

### 4.3. Characterization of the Hydrogels

#### 4.3.1. Rheological Characterization

The rheological behavior of both sterilized and non-sterilized HGs was assessed one week (T0), two weeks (T1) and one month (T2) after preparation, using a Kinexus lab+ rotational rheometer (Malvern Instruments Ltd., Worcestershire, UK) with a plate-plate geometry and a working gap of 1.0 mm between the plates. After removing the excess sample, the viscosity (Pa·s) and shear stress (Pa) of each HG were measured while increasing the shear rate from 0.1 to 100.0 s^−1^ (10 samples per decade) at 25 °C.

Afterward, the most promising HGs were evaluated regarding thixotropy by performing a three-step shear test in the Kinexus lab+ rheometer. At first, a low shear rate of 0.1 s^−1^ was applied for 60 s to imitate the at-rest state of the HGs. Then, a shear rate of 100 s^−1^ was employed in stage two for 30 s to replicate the breakdown of the HG structure under high shear. In the third stage, to observe the recovery of the initial viscosity, the shear rate was reduced to 0.1 s^−1^ and held for 600 s, and then the recovery rate (%) was calculated [54].

The rSpace for Kinexus software (version 2.0.0.0, NETZSCH-Gerätebau GmbH, Selb, Germany) collected and processed the rheological data.

#### 4.3.2. Texture Analysis

A texture analyzer (TA-XT2i, Stable Micro Systems, Surrey, UK) with a 5 kg load cell was used to evaluate the mechanical properties of both sterilized and non-sterilized HGs one, two and four weeks after preparation. For each measurement, the HG sample was compressed with a cylindrical probe (diameter 25 mm, P/25L) at a test velocity of 3 mm/s. When a trigger force of 0.049 N was achieved, the probe penetrated the sample to a depth of 5 mm and then returned to the starting position.

The Texture Exponent 32 software (version 6.1.26.0, Stable Micro Systems, Surrey, UK) was used to determine some relevant texture properties (firmness and adhesiveness) by plotting the resulting force vs. distance. Whereas firmness is given by the maximum force (N), the negative area (N·mm) is related to adhesiveness, corresponding to the work needed to separate the probe from the sample after penetration [55,56]. Measurements were performed in triplicate for each HG at 25 °C and the results are presented as mean ± standard deviation (SD).

#### 4.3.3. pH Measurement

The pH value of the different HGs was measured using a pH meter (HI2211 Basic pH/ORP/°C Meter, HANNA Instruments Inc., Woonsocket, RI, USA) one, two and four weeks after preparation. Each measurement was taken three times at room temperature and all data are presented as mean ± SD.

#### 4.3.4. Sol-Gel Transition

The sol-gel-phase transition behavior (i.e., gelation) of the aqueous PF127 dispersions was studied by performing oscillatory tests in the Kinexus lab+ rheometer.

First, an amplitude sweep test was conducted to identify the linear viscoelastic (LVE) region, where the HG structure remains intact. This preliminary test was carried out in the shear strain range of 0.1–100.0% (10 samples per decade) at 25 °C and a constant frequency of 1 Hz. Then, the gelation temperature was investigated within the LVE region (0.5% deformation) by increasing the temperature from 4 to 50 °C at a heating rate of 5 °C/min with a 1 Hz frequency. To determine gelation time, time sweeps up to 10 min were performed at a fixed temperature of 25 °C with a 1 Hz frequency and 0.5% strain [57]. For both temperature and time sweep tests, the lower plate was pre-cooled to 4 °C before placing the aqueous dispersions of PF127. The following viscoelastic parameters were recorded: elastic modulus (G′), viscous modulus (G″), and phase angle. The G′/G″ crossover point indicated the gelation point [58].

#### 4.3.5. Fourier-Transform Infrared (FT-IR) Spectroscopy

FT-IR analysis was performed to elucidate the sterilization-induced changes in the chemical structure of the HGs. Each sterilized and non-sterilized HG was placed on a PerkinElmer Frontier™ FT-IR spectrometer (Waltham, MA, USA) equipped with a diamond attenuated total reflectance system. Water was removed from the samples using a hairdryer set on low heat. The spectra were obtained by collecting 16 scans between 4000 and 600 cm^−1^ with a resolution of 4 cm^−1^, using the PerkinElmer Spectrum™ 10 software.

#### 4.3.6. Differential Scanning Calorimetry (DSC)

DSC measurements were carried out to understand whether the elevated temperature in steam sterilization affected the physical properties of the HGs, using a DSC 214 Polyma^®^ equipment (NETZCH-Gerätebau GmbH, Selb, Germany) calibrated with pure indium (melting point: Tm = 156.6 °C, heat of fusion: ΔfH = 28.45 J/g). Briefly, the sterilized and non-sterilized HGs as well as the polymers (0.5–15 mg) were weighted into aluminum crucibles. In the case of HGs, the crucibles were placed on a hot plate at 40 °C to evaporate the water before sealing. An empty crucible was used as a reference. All samples were scanned from 25 to 200 °C at a rate of 10 °C/min, under a nitrogen atmosphere (flow rate 50 mL/min). DSC data were obtained using the Proteus^®^ software (version 8.0.1, NETZCH-Gerätebau GmbH, Selb, Germany).

#### 4.3.7. Statistical Analysis

The results of texture analysis and pH measurement were presented as mean ± SD (*n* = 3) and statistically analyzed using the IBM SPSS Statistics software for Windows (version 28.0, IBM Corp., Armonk, NY, USA). After investigating normality and homogeneity of variance with Shapiro–Wilk and Levene’s test, a non-parametric Kruskal–Wallis’ test for independent samples was performed. A *p*-value below 0.05 was considered statistically significant.

## Figures and Tables

**Table 1:** Composition of the developed HGs (% *w*/*w*).

Composition	Hydrogels		
	C940 HG	PF127 HG	SA HG
Carbopol^®^ 940	0.5	-	-
Pluronic^®^ F-127	-	20.0	-
Sodium alginate	-	-	2.0
Fenonip^®^	0.1	0.1	0.1
Triethanolamine	q.s.	-	-
Purified water	q.s. 100	q.s. 100	q.s. 100

## Data Availability

The data supporting the reported results are included in the article, and the raw data from oscillatory rheological measurements are available upon request.
